# A novel S1P analogue/myosin light chain kinase inhibitory peptide-encargoed nanocarrier to attenuate lung vascular leak

**DOI:** 10.1093/ajrcmb/aanag069

**Published:** 2026-03-27

**Authors:** Saad Sammani, Tadeo Bermudez, Carrie L Kempf, Nancy G Casanova, Belinda L Sun, Jin H Song, Annie Hernandez, Haifei Xu, Jason Canizales Galaviz, Xiaoguang Sun, Steven M Dudek, Nathan H Johnson, Sara M Camp, Christopher Fox, Robert Kinsey, Stan Miele, Donna D Zhang, Christian Bime, Aikseng Ooi, Joe G N Garcia

**Affiliations:** Departments of Medicine and Pathology, University of Arizona Health Sciences, Tucson, AZ, United States; Departments of Medicine and Pathology, University of Arizona Health Sciences, Tucson, AZ, United States; Center for Inflammation Science and Systems Medicine, University of Florida Herbert Wertheim Scripps Research Institute, Jupiter, FL, United States; Center for Inflammation Science and Systems Medicine, University of Florida Herbert Wertheim Scripps Research Institute, Jupiter, FL, United States; Departments of Medicine and Pathology, University of Arizona Health Sciences, Tucson, AZ, United States; Center for Inflammation Science and Systems Medicine, University of Florida Herbert Wertheim Scripps Research Institute, Jupiter, FL, United States; Center for Inflammation Science and Systems Medicine, University of Florida Herbert Wertheim Scripps Research Institute, Jupiter, FL, United States; Center for Inflammation Science and Systems Medicine, University of Florida Herbert Wertheim Scripps Research Institute, Jupiter, FL, United States; Center for Inflammation Science and Systems Medicine, University of Florida Herbert Wertheim Scripps Research Institute, Jupiter, FL, United States; Departments of Medicine and Pathology, University of Arizona Health Sciences, Tucson, AZ, United States; Department of Medicine, University of Illinois at Chicago, Chicago, IL, United States; Center for Inflammation Science and Systems Medicine, University of Florida Herbert Wertheim Scripps Research Institute, Jupiter, FL, United States; Center for Inflammation Science and Systems Medicine, University of Florida Herbert Wertheim Scripps Research Institute, Jupiter, FL, United States; Infectious Disease Research Institute, Seattle, WA, United States; Infectious Disease Research Institute, Seattle, WA, United States; Aqualung Therapeutics Corp., Tucson, AZ, United States; Center for Inflammation Science and Systems Medicine, University of Florida Herbert Wertheim Scripps Research Institute, Jupiter, FL, United States; Departments of Medicine and Pathology, University of Arizona Health Sciences, Tucson, AZ, United States; Center for Inflammation Science and Systems Medicine, University of Florida Herbert Wertheim Scripps Research Institute, Jupiter, FL, United States; Division of Hematology and Oncology, Department of Medicine, University of Florida, Gainesville, FL, United States; Center for Inflammation Science and Systems Medicine, University of Florida Herbert Wertheim Scripps Research Institute, Jupiter, FL, United States

**Keywords:** NTyP-100, ARDS, PIK, TySIP, nanocarrier

## Abstract

An unremitting increase in lung vascular permeability is the pathophysiological hallmark of acute lung injury (ALI) and drives both severity and mortality. Therapies with the capacity to quickly restore vascular integrity in ALI remains a serious unmet need. Our laboratory was first to report both the vascular barrier-protective effects of sphingosine-1-phosphate (S1P) and S1P analogues, such as tysiponate (TySIP), and the efficacy of peptide inhibitors (PIK) of non-muscle myosin light chain kinase (nmMLCK) as dual complementary strategies to reduce vascular permeability. The current study evaluates a novel nanocarrier (NTyP-100) containing conjugated TySIP and encargoed PIK as a pharmacologic approach to vascular barrier restoration in rodent models of LPS-induced ALI. NTyP-100 (or control) was delivered intravenously to wild-type C57BL/6J mice exposed to a “one-hit” lipopolysaccharide (LPS; 18 hours) ALI model or to Sprague–Dawley rats challenged by a “2-hit” ALI model combining LPS (18 hours) and exposure to high tidal volume mechanical ventilation (4 hours). Compared to TySIP or PIK alone, intravenous NTyP-100 produced the highest reduction (∼40%) in inflammatory injury in murine and rat ALI models (hematoxylin and eosin, immunohistochemistry p-MLC staining, BAL cells), with marked reductions in vascular leak (Evan Blue Dye leakage, BAL protein) and biochemical indices of inflammation. Genomic studies underscored NTyP-100 attenuation of ALI-mediated dysregulated barrier-regulatory signaling pathways (inflammatory response, innate immunity, TNF, IL-17, apoptosis). These studies demonstrate the successful therapeutic targeting of vascular barrier properties and supports the NTyP-100 nanocarrier as a strategy to address the unmet need for novel therapeutics that mitigate inflammatory injury and vascular permeability.

## Introduction

Vascular leakage is the pathophysiologic hallmark of acute lung injury (ALI) and the acute respiratory distress syndrome (ARDS),^1^ severe critical care inflammatory illnesses with mortality rates >30%.^2^ Increased vascular permeability is also a clinically important feature of chronic inflammatory diseases such as asthma, arthritis, and autoimmune disorders.^3^ Despite advances in the understanding of ALI/ARDS pathobiology and the recognition of the contribution of mechanical ventilation–induced mechanical stress to unremitting increases in lung vascular permeability, US Food and Drug Administration (FDA)–approved pharmacotherapies that limit vascular leak and restore vascular integrity remain an enormous unmet medical need. We were the first to report the capacity for sphingosine-1-phosphate (S1P), an endogenous circulating phospholipid released from multiple cells including endothelial cells (ECs) and platelets,^4^ as a novel angiogenic factor^5^ that profoundly enhances/restores lung EC barrier function via ligation of the S1P1 receptor (S1PR1).^6^ Limiting the potential clinical utility of S1P as a barrier-protective therapeutic include adverse S1P clinical effects (bradycardia, increased airway hyperresponsiveness) due to engagement of other S1P receptors such as S1PR3^7-10^ and the induction of β-arrestin recruitment, S1PR1 ubiquitination, and subsequent S1PR1 proteasomal degradation by S1P and several S1P analogues, including SEW-2871 (5-[4-Phenyl-5-(trifluoromethyl)thiophen-2-yl]-3-[3-(trifluoromethyl)phenyl]1,2,4-oxadiazole) and FTY-720 (2-amino-2-[2-(4-octylphenyl) ethyl]propane-1,3-diol).^11^^,^^12^ The ensuing decreases in S1PR1 availability limit lymphocyte trafficking, creating an immunosuppressive environment but potentially exacerbating ALI/ARDS vascular leak and thus disease severity.^8^^,^^13-16^ These observations led us to prioritize a phosphonated FTY-720 analogue, tysiponate (TySIP), as a preferred S1P analogue due to significant efficacy in enhancing EC barrier integrity, both in vitro and in vivo, without S1PR1 protein degradation or enhanced immunosuppression.^11^^,^^16^^,^^17^

In addition to the involvement of the S1P/S1PR1 signaling pathway in EC barrier regulation, we have also shown that the non-muscle myosin light chain kinase isoform (nmMLCK), a central regulator of EC cytoskeletal contractile properties,^18^ is intimately involved in the regulation of vascular inflammatory responses including neutrophil diapedesis^6^ as well inflammation-mediated increases in vascular permeability.^19-23^ We previously demonstrated that nmMLCK is a druggable inflammatory target as intravenous injection of a membrane-permeant peptide inhibitor of nmMLCK (PIK) or nmMLCK-silencing RNAs produced dose-dependent attenuation of LPS- and ventilation-induced lung injury with reductions in alveolar and vascular permeability and leukocyte influx.^21^

With this background, the present study was designed to evaluate a novel therapeutic nanocarrier (NTyP-100) as a TySIP delivery vehicle for vascular targeting with the nmMLCK inhibitory peptide, PIK, as cargo. Compared to either TySIP or PIK alone, intravenously (IV) delivered NTyP-100 produced the highest reduction in ALI severity (∼40%) in murine and rat models of inflammatory lung injury as determined by lung tissue hematoxylin and eosin (H&E), levels of myosin light chain phosphorylation (p-MLC), BAL cells/protein, plasma cytokines, Evans blue dye (EBD) leakage studies, and both biochemical and genomic indices. These studies demonstrate the successful targeting of vascular barrier properties and support the NTyP-100 nanocarrier as a strategy to address the unmet need for novel therapeutic strategies that mitigate inflammatory injury and vascular permeability.

## Materials and methods

### Reagents

Reagents were purchased from MilliporeSigma (St. Louis, MO, USA) unless otherwise noted. TySIP was generated as previously described.^24^ PIK (GenScript, Piscataway, NJ, USA), Dipalmitoyl­phosphatidylcholine (DPPC, Lipoid LLC, Newark, NJ, USA), DSPE-PEG2000 (Corden Pharma, Liestal, Switzerland), and cholesterol (Wilshire Technologies, Princeton, NJ, USA) were commercially obtained and NTyP-100 was generated by the Infectious Disease Research Institute (Seattle, WA, USA).

### Nanocarrier generation

DPPC, DSPE-PEG2000, cholesterol, TySIP, and PIK were added with chloroform; the flask was warmed to 60 °C and sonicated (2 hours), producing nanocarriers (NCs; average 106 nm diameter by dynamic light scattering). NCs were sterile filtered and aseptically filled into sterile glass vials.

### Animal studies

Animal studies were performed via University of Arizona subcontract approved by the University of Arizona Institutional Animal Care and Use Committee. A “one-hit” lipopolysaccharide (LPS) murine model of ALI^25^ employed C57BL/6J male mice (8-12 weeks of age, The Jackson Laboratory, Bar Harbor, ME, USA) (*n* = 5/group) anesthetized with intraperitoneal (IP) ketamine (100 mg/kg)/xylazine (10 mg/kg). Intratracheally (IT) intubated mice received LPS (*Escherichia coli* 0127:B8, 1 mg/kg, 40 μL) or PBS (40 μL). TySIP, PIK, and NTyP-100 were injected IV 10 minutes after IT LPS instillation. Mice were again anesthetized 18 hours later prior to sacrifice by exsanguination.^25^^,^^26^ A “2-hit” LPS/Ventilator Induced Lung Injury (VILI) rat model of ALI^27^ employed male Sprague–Dawley rats (300-350 g, Charles River, Wilmington, MA, USA), (*n* = 5/group) anesthetized via IP ketamine/xylazine. IT-intubated rats received LPS (0.1 mg/kg) or PBS. Eighteen hours later, rats were reanesthetized and mechanically ventilated (MV) for 4 hours with room air, tidal volume (V_T_) = 20 mL/kg, respiratory rate = 70 breaths/minute, and positive end-expiratory pressure = 0 cm H_2_O (VentElite, Harvard Apparatus, Holliston, MA, USA).^2^ NC-TySIP, NC-PIK, NTyP-100, or PBS were injected IV 10 minutes post–LPS instillation.

### Bronchoalveolar lavage analysis

At the end of the study, BAL fluid was collected and cells differential counts were obtained as previously described.^27^ BAL supernatants were processed for total protein measurements as previously described.^27^

### Histology and immunohistochemistry analyses

Paraffin-embedded lung tissues with H&E staining and immunohistochemistry (IHC) studies for p-MLC detection (rabbit anti-phospho-Ser19, Invitrogen, Waltham, MA, USA), were analyzed and quantified utilizing ImageJ software as previously described.^27^

### Evans blue dye evaluation

“Two-hit” model rats and controls were intravenously injected with EBD (30 mg/kg) 1 hour before harvesting.^20^^,^^28^ Lungs were perfused for blood removal and whole lung images collected.

### Biochemical analyses

Western blotting of lung tissue homogenates with densitometric quantification was performed as previously described.^25^^,^^27^^,^^29^

### Lung tissue RNA sequencing analysis

Bulk RNA sequencing (RNA-seq) of rat lung tissue RNA was performed and analyzed as previously reported.^30^^,^^31^ HISAT was used to align clean reads to the reference genome. DEseq2^32^ algorithms were used to detect statistically significant differentially expressed genes (DEGs). A false discovery rate (FDR) of 0.05 was applied.^32^ Gene Ontology (GO) and Kyoto Encyclopedia of Genes and Genomes (KEGG) databases identified significantly enriched pathways.

### Statistical analyses

Continuous data were compared using nonparametric methods, t-test for means from different experimental groups, categorical data by χ^2^ test, and standard one-way ANOVA and groups using the Tukey test (*P* <.05).^27^^,^^29^

## Results

### Protective effect of the NTyP-100 nanocarrier in reducing LPS-induced murine lung injury

H&E lung tissue staining from wild-type C57BL/6J mice exposed to the “one-hit” LPS model (1 mg/kg IT, 18 hours) showed substantial inflammatory lung injury characterized by marked neutrophil lung infiltration, increased alveolar wall thickness, and interstitial/alveolar edema compared to control PBS-treated mice (Figure 1A). These results were substantiated by quantification of the percentage of H&E-positive areas measured by ImageJ software (Figure 1B). Consistent with the histologic evidence of inflammatory lung injury, LPS-exposed mice exhibited marked increases in BAL inflammatory cell counts (Figure 1C) and elevated BAL total protein concentrations (Figure 1D) compared to PBS-treated control mice (*P* <.05 vs controls).

**Figure 1:**
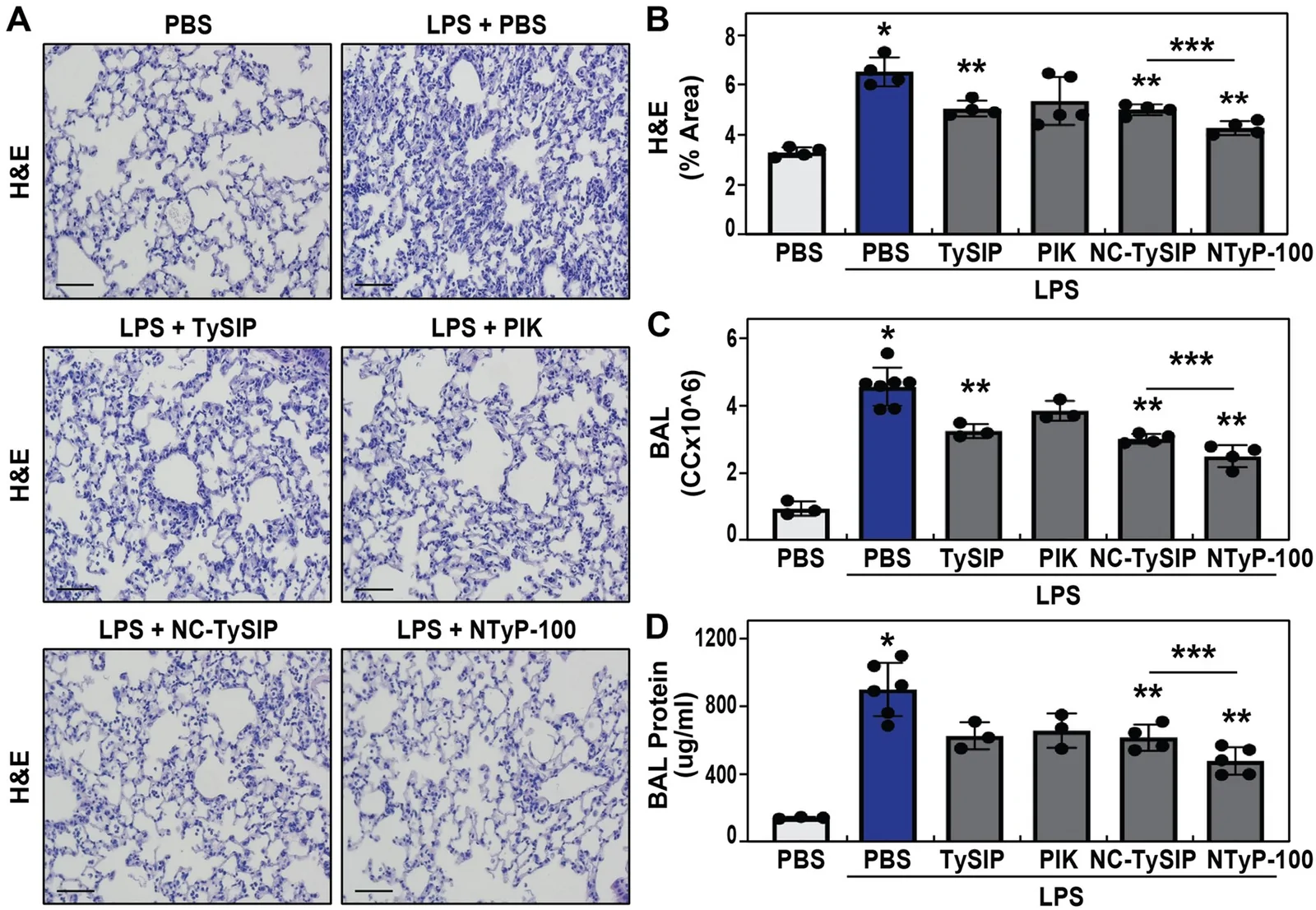
Synergistic protection by NTyP-100 nanocarrier components in LPS-challenged mice. (A) H&E staining of lung tissues from PBS-treated C57BL/6J, 10- to 12-week-old mice indicates dramatic interstitial and alveolar inflammation with marked neutrophil infiltration and alveolar edema compared to control mice (insets). Substantial attenuation of the inflammatory lung injury observed was exhibited in mice receiving either TySIP (1.5 μg/kg), the nanocarrier conjugated to TySIP (NC-TySIP) (1.5 μg/kg), PIK (0.4 mg/kg), or NTyP-100, with the highest level of attenuation observed in NTyP-100–treated mice. (B) Histologic quantification (ImageJ) revealed significantly increased % of H&E-positive areas in LPS-induced lung injury compared to control mice (*P <.05). Histologic injury was significantly decreased in mice that received either TySIP (1.5 μg/kg), PIK (0.4 mg/kg), NC-TySIP (1.5 μg/kg), or NTyP-100 (0.26 mg/kg) delivered intravenously 1 hour after LPS challenge (**P <.05, compared to PBS-treated mice). The strongest attenuation of lung injury was observed in mice receiving NTyP-100 (***P <.05). (C, D) BAL inflammatory total cell counts and BAL total protein concentrations were significantly increased in LPS-challenged mice treated with PBS compared to controls (*P <.05). LPS-challenged mice receiving TySIP (1.5 μg/kg), PIK (0.4 mg/kg), NC-TySIP (1.5 μg/kg), or NTyP-100 (0.26 mg/kg) (**P <.05) showed significant attenuation compared to PBS-treated mice, with greatest attenuation of BAL indices observed in mice that received NTyP-100 (**, ***P <.05). Abbreviations: H&E, hematoxylin and eosin; LPS, lipopolysaccharide; NC, nanocarrier; PBS, phosphate-buffered saline; TySIP, tysiponate.

The intravenous administration of either TySIP (1.5 μg/kg), PIK (0.4 mg/kg), NC-TySIP (1.5 μg/kg), or NTyP-100 (0.26 mg/kg), delivered 10 minutes post–LPS challenge, substantially attenuated the severity of inflammatory lung injury reflected by reductions in leukocyte infiltration and interstitial/alveolar edema (Figure 1A, B), and by reduced BAL cell counts and total protein levels (Figure 1C, D) compared to LPS-PBS–challenged mice. Among the therapeutic agents utilized, NTyP-100 (0.26 mg/kg) provided the greatest attenuation of lung injury as determined by H&E histology (Figure 1A, B), BAL cell counts (Figure 1C), and BAL total protein levels (Figure 1D).

### Protective effect of NTyP-100 in reducing LPS/VILI-induced rat lung injury

H&E lung tissue staining from Sprague–Dawley rats exposed to a “2-hit” LPS/VILI rat ARDS model (LPS/high V_T_ MV) revealed substantial inflammatory lung injury characterized by a marked neutrophil infiltration, increased alveolar wall thickness, and interstitial/alveolar edema compared to control LPS/VILI-PBS–challenged rats (Figure 2A). This was substantiated by quantification of the percentage of H&E-positive area measured by ImageJ software (Figure 2B) and marked increases in both BAL inflammatory cell counts and total protein concentrations (Figure 2C, D) as compared to LPS/VILI-PBS–challenged rats (*P* < .05, vs controls). Intravenous administration of NC-TySIP (1.5 μg/kg) or NC-PIK (0.4 mg/kg), delivered 1 hour after LPS/VILI challenge, substantially reduced the severity of inflammatory lung injury reflected by reductions in leukocyte infiltration and interstitial/alveolar edema (Figure 2A, B), and reduced BAL cells/total protein levels (Figure 2C, D). Among the therapeutic agents utilized, NTyP-100 (0.26 mg/kg) provided the greatest mitigation of lung injury severity reflected in H&E histology (Figure 2A, B) and BAL cells/protein (Figure 2C, D).

**Figure 2:**
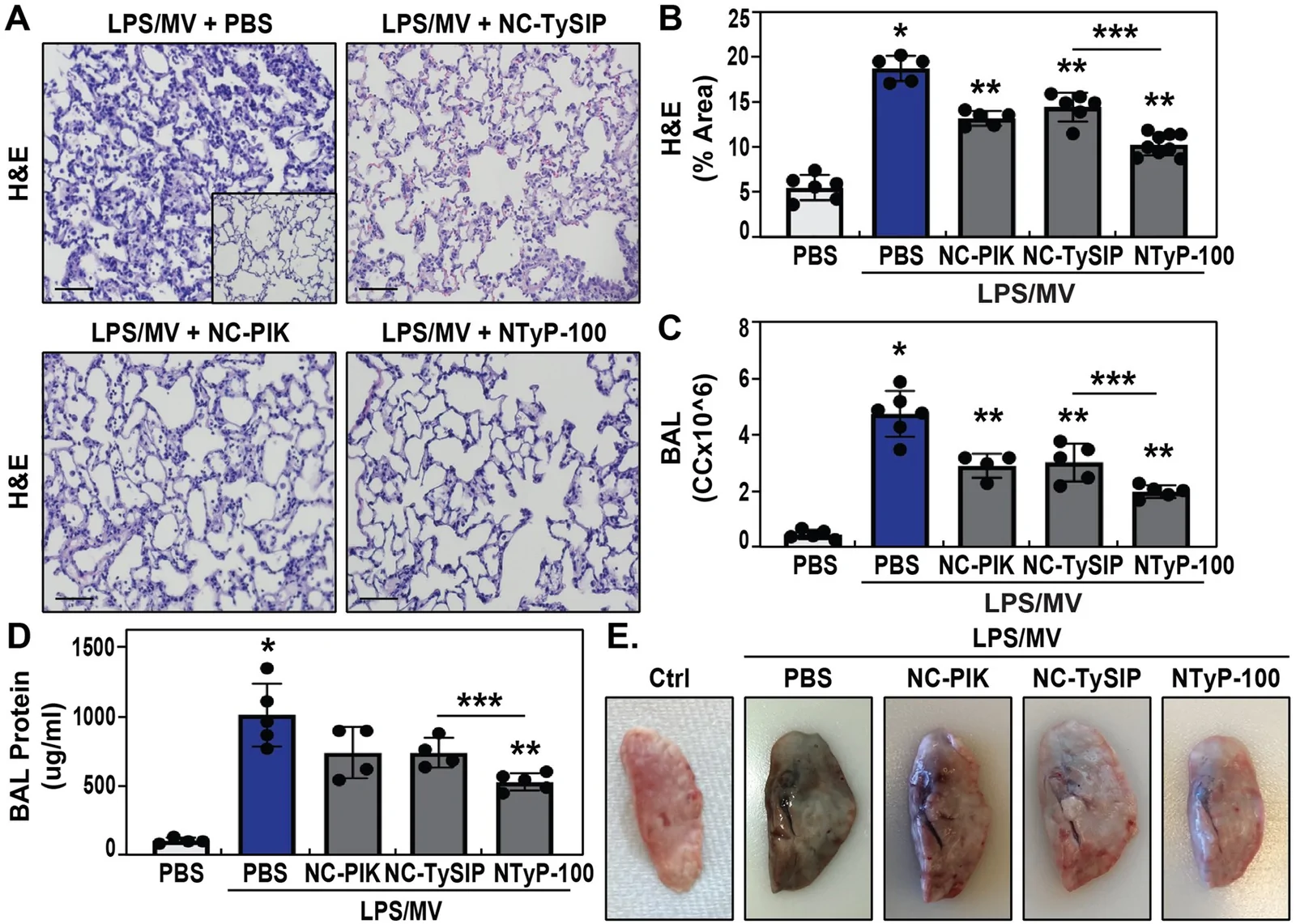
Synergistic protection by NTyP-100 nanocarrier components in the LPS/VILI rat lung injury model. (A) H&E staining of LPS/VILI-challenged rat lung tissues indicates dramatic interstitial and alveolar inflammation with marked neutrophil infiltration and alveolar edema compared to control rats (insets). Substantial attenuation in the inflammatory lung injury was observed in rats receiving either N-PIK (0.4 mg/kg), NC-TySIP (1.5 μg/kg), or NTyP-100 (0.26 mg/kg) 10 minutes after LPS challenge, with the highest level of attenuation observed in the NTyP-100–treated rats. (B) Histologic ImageJ quantification of % H&E-positive area in LPS/MV-induced histologic lung injury indicates a significant increase in PBS-treated rats compared to controls (*P <.05) with significant attenuation in rats receiving either NC-PIK (0.4 mg/kg), NC-TySIP (1.5 μg/kg), or NTyP-100 (0.26 mg/kg) 1 hour after LPS challenge, with the highest level of attenuation observed in the NTyP-100–treated group (*n* = 5-6/group, **, ***P <.05 for NTyP-100 vs NC-PIK or NC-TySIP). (C, D) BAL inflammatory total cell counts and BAL total protein concentration were significantly increased in LPS/VILI rats treated with PBS compared to controls (*P <.05) with substantial attenuation in rats receiving either NC-PIK (0.4 mg/kg), NC-TySIP (1.5 μg/kg), or NTyP-100 (0.26 mg/kg) 1 hour after LPS challenge. The highest level of attenuation was observed in the NTyP-100–treated group (*n* = 5-6/group, **, ***P <.05 for NTyP-100 vs NC-PIK or NC-TySIP). (E) Images obtained from the excised whole left lung of rats that were injected with EBD (30 mg/kg) 2 hours before harvesting. Images demonstrate a prominent EBD vascular extravasation in the PBS-treated LPS/VILI-exposed group compared to control rats. In contrast, intravenous injection of either NC-PIK, NC-TySIP or NTyP-100 reduced EBD vascular extravasation in lung tissue, with the strongest diminishment in EBD color observed in the NTyP-100–treated group. Abbreviations: EBD, Evans blue dye; H&E, hematoxylin and eosin; LPS, lipopolysaccharide; MV, mechanical ventilation; NC, nanocarrier; PBS, phosphate-buffered saline; TySIP, tysiponate; VILI, Ventilator Induced Lung Injury.

The effects of these therapeutic agents on lung vascular leakage in LPS/VILI-exposed rats was directly assessed by evaluating EBD vascular extravasation. The most prominent reduction in EBD vascular extravasation in LPS/VILI-exposed rats was observed in rats receiving NTyP-100 effects (0.26 mg/kg) as compared to either NC-TySIP (1.5 μg/kg) or NC-PIK (0.4 mg/kg). These studies are consistent with synergistic barrier enhancement by TySIP and PIK components of the NTyP-100 nanocarrier (Figure 2E).

### Validation of nmMLCK inhibition by the PIK-containing NTyP-100 nanocarrier

We next verified the capacity of the NTyP-100 nanocarrier to reduce nmMLCK-mediated p-MLC proteins by employing IHC staining of p-MLC in rat lung tissues. Figure 3A and B depict the increased p-MLC immunoreactivity (*P* <.01, controls in inset) in lung tissues from LPS/VILI-challenged rats. LPS/VILI-challenged rats receiving either NC-PIK or NTyP-100 demonstrated significant decreases in p-MLC immunoreactivity (*P* <.01) whereas rats receiving NC-TySIP demonstrated minimal influences on p-MLC immunoreactivity (Figure 3A, B). Western blot analysis of lung homogenates for p-MLC immunoreactivity with densitometric quantification supported the IHC studies with substantial levels of p-MLC immunoreactivity in lung tissue from LPS/VILI-challenged rats and greatest reduction observed in lung tissues from NTyP-100–treated rats, substantial reduction in NC-PIK rat lung tissues, and no reduction in lung tissues from NC-TySIP–treated rats (*P* <.01) (Figure 3C, D; Figure S1).

**Figure 3:**
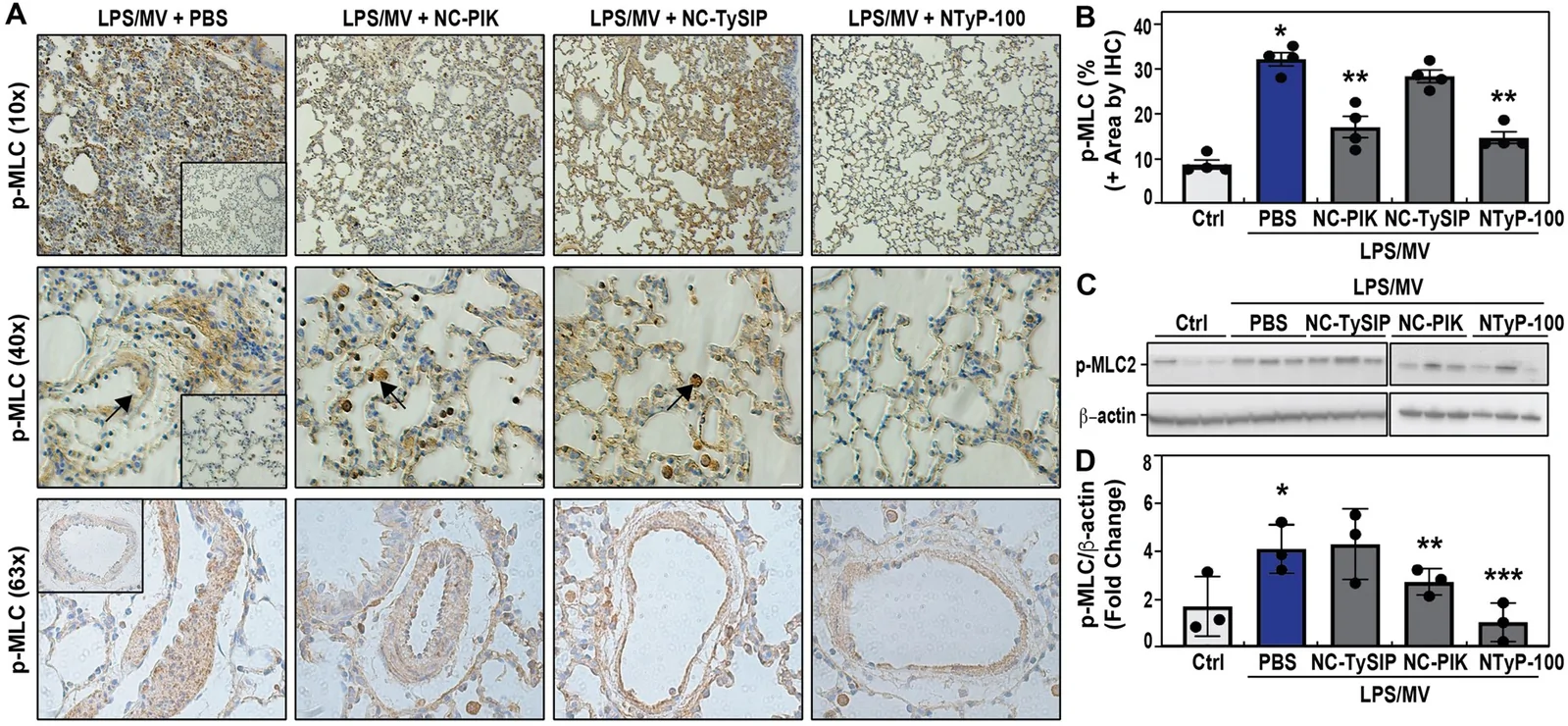
Effects of NTyP-100 on LPS/VILI-induced MLC phosphorylation in lung tissues. (A) Levels of p-MLC in rat lungs were detected by IHC staining with p-MLC antibodies (rabbit anti-phospho-MYL9 [Ser19] polyclonal IgG, PIPA517726, Invitrogen) at 1:100 dilution, and biotinylated secondary antibodies. IHC staining of LPS/VILI-exposed lung tissues demonstrated marked increases in p-MLC immunoreactivity (brown), compared with unexposed control rats (magnificent ×100, ×400). LPS/VILI-exposed rats receiving NC-PIK or NTyP-100 demonstrated dramatically decreased p-MLC immunoreactivity compared to PBS-treated controls. (B) Quantification of IHC images by ImageJ software confirmed significantly expanded p-MLC immunoreactivity in LPS/VILI-exposed rats compared to PBS-treated controls with significant reduction of p-MLC immunoreactivity in NC-PIK– and NTyP-100–treated rats (*, ** *P* <.01). (C, D) p-MLC immunoreactivity was also assessed by Western blot of lung tissue homogenates with band densitometry quantification revealing significant increases in lung p-MLC phosphorylation in LPS/MV-exposed lungs compared to controls and greatest reduction in the levels of p-MLC immunoreactivity in NTyP-100–treated rats, substantial reduction in NC-PIK, rats and no reduction in NC-TySIP–treated rats (*, **, ***P < .01). Abbreviations: Ctrl, control; IHC, immunohistochemistry; LPS, lipopolysaccharide; MLC, myosin light chain; MV, mechanical ventilation; NC, nanocarrier; PBS, phosphate-buffered saline; p-MLC, myosin light chain phosphorylated proteins; TySIP, tysiponate; VILI, Ventilator Induced Lung Injury.

### NTyP-100 reduces biochemical indices of LPS/VILI-induced lung inflammation

We next evaluated protein parameters of inflammatory lung injury by Western blot analysis of lung tissue homogenates from LPS/VILI-challenged rats combined with densitometric quantification. Figure 4 depicts the marked increases in levels of phosphorylated NF-κB and ERK1/2 and p38 MAP kinases, and increased expression of NAMPT, an immune effector that is a TLR4 ligand and DAMP.^33^ Together, these results reflect significant innate immunity activation and in each case, increases in inflammatory protein expression were reduced in LPS/VILI-challenged rats receiving NC-PIK or NTyP-100. Whereas reduced NAMPT expression was comparable between NC-PIK and NTyP-100 (Figure 4E), reductions in phosphorylated NF-κB, ERK1/2, and p38 MAP were differentially reduced by NTyP-100 treatment (Figure 4B-D).

**Figure 4:**
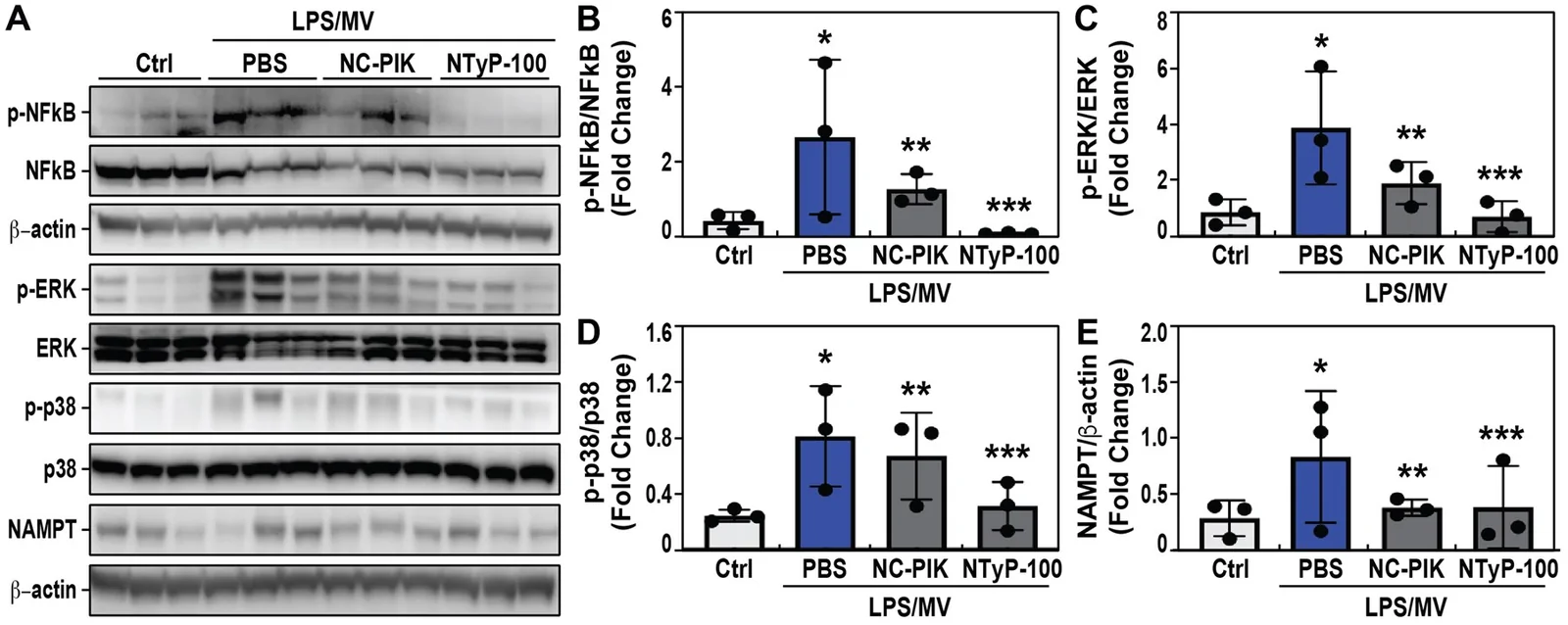
Attenuation of biochemical indices of LPS/VILI-induced inflammatory signaling in NTyP-100–treated rats. (A, B) Representative Western blots showing the increased phosphorylation of NF-κB in lung tissues from LPS/MV-challenged rats treated with PBS, NC-PIK, or NTyP-100 compared with controls. Marked increase in NF-κB phosphorylation with band densitometry quantification was observed in LPS/VILI-challenged lung homogenates consistent with inflammatory lung injury. Rats receiving NC-PIK showed partial inhibition of NF-κB activation whereas rats receiving NTyP-100 displayed profound suppression of NF-κB phosphorylation. (C, D) Similar to NF-κB phosphorylation, Western blot studies demonstrated marked increases in ERK and p38 MAPK phosphorylation/activation in LPS/VILI-challenged lung homogenates quantified by densitometric measurements. (E) Western blot studies depicting increased levels of the DAMP and innate immunity effector, NAMPT,^27^^,^^29^ in LPS/VILI-challenged lung homogenates by NAMPT immunoreactivity with densitometric measurements. Rats receiving either NC-PIK or NTyP-100 displayed reduced NAMPT immunoreactivity. (*P <.05 compared with control, **P <.05 compared to LPS/MV-PBS, ***P <.01 compared to LPS/MV-PBS). Abbreviations: Ctrl, control; LPS, lipopolysaccharide; MV, mechanical ventilation; NC, nanocarrier; PBS, phosphate-buffered saline; VILI, Ventilator Induced Lung Injury.

### NTyP-100 reduces LPS/VILI-induced inflammatory gene/pathway dysregulation

The comparison of DEGs between the control and LPS/VILI groups identified a total of 535 DEGs (FDR <0.05 Fold Change (FC2)) with top upregulated DEGs that included *ilr2* (interleukin 1 receptor type 2), *Osmr* (oncostatin-M receptor), and *Nifl3* (nuclear factor, interleukin 3) and significant downregulation of *Slc5a1*, a solute carrier family 5 member (Figure 5A). A heatmap of the 79 DEGs identified in the comparison of LPS/VILI-exposed PBS- and NTyP-100–treated rats (FDR <0.1) included multiple upregulated DEGs (*Tbck*, *Reg3g*, *TRIM45*, *CXCR1*) and downregulated DEGs (*S100A11*, *reg3g*, *edil3*, *serpinb7*, *Gja5*, *Hspb1*, *Il17rc Tpd52l*), associated with either inflammatory or barrier-regulatory pathways (Figure 5B; Figure S2). Enrichment KEGG pathway analysis confirmed “cytokine–cytokine receptor interaction” as the most significant LPS/VILI pathway (FDR 3.18E-09), a finding consistent with previous reports in this LPS/VILI model.^29^ Additional overrepresented inflammation-related pathways included “viral protein interaction with cytokine,” and “IL-17 signaling” (Figure 5C, D; Figure S3; Table 1). Further comparison of the number of genes annotated to specific dysregulated KEGG pathway categories (LPS/VILI PBS vs NTyP-100) showed NTyP-100 to significantly decrease the number of dysregulated genes contained in each pathway, with the majority of genes linked to human disease, metabolism, and innate immunity. Finally, analysis of GO biological terms highlighted the “inflammatory response,” “response to hyperoxia,” “reactive oxygen species [ROS] regulation,” “upregulation of fibroblast proliferation,” and “positive regulation of NF-kB transcription” as top biological processes (Figure S4A). The NTyP-100–treated rats exhibited an attenuation of NF-κB–driven gene upregulation observed in LPS/VILI-PBS–treated rats (Figure S4B) and a reduction in the number of dysregulated fibrosis-related genes (from 39 to 23 DEGs) and dysregulated genes associated with fibroblast activation (*Pdgfc*, *Ndufs4*, *Rps3a*, *Rasgrf1*, *Fbln1*, *Wnt5a*, *Tgif1*, *Agt*, *Pdgfrb*, *Esr1*, *Tgfb1*, *Cdk4*, *Fbrs*, *Abl1*, *Kmt2c*, *Ager*, *Ngfr*, *Pml*, *Anxa2*) (Figure S4C).

**Figure 5:**
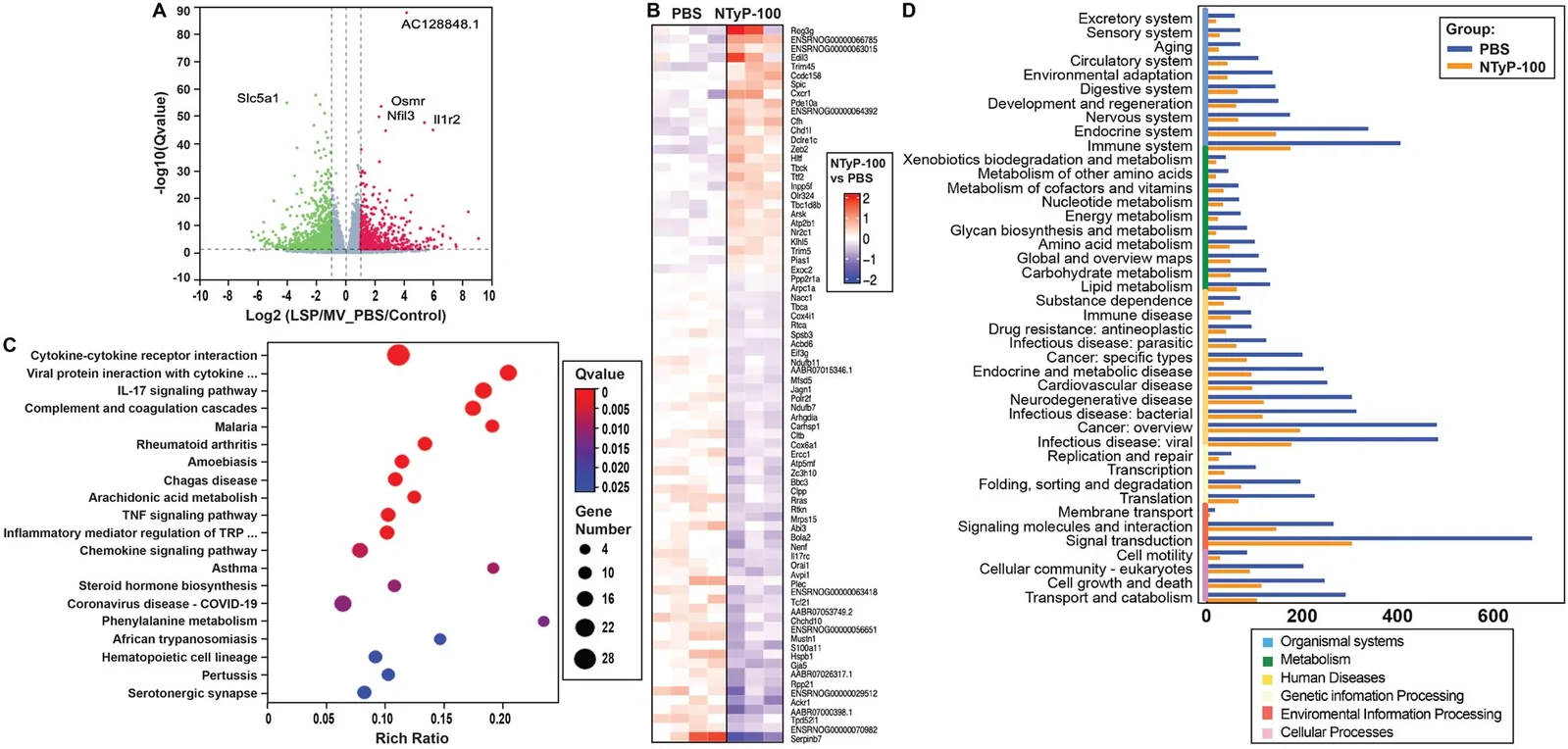
Genomic influence of NTyP-100 in LPS/MV-exposed lung tissues. (A) Volcano plot shows DEGs in the comparison of LPS/VILI-exposed rats vs control, unexposed rats. The x-axis represents the fold change of the difference after conversion to log2, and the y-axis represents the significance value after conversion to -log_10_. Red represents upregulated DEGs; green downregulated DEGs. The top upregulated DEGs included *ilr2* (interleukin 1 receptor type 2), *Osmr* (oncostatin-M receptor), and *Nifl3* (nuclear factor, interleukin 3) with significant downregulation of *Slc5a1*, a solute carrier family 5 member. (B) Heatmap of the 79 DEGs identified in comparison of LPS/VILI to LPS/VILI-exposed, NTyP-100–treated rats (FDR <0.1). Increasing expression levels are reflected by increasing color warmth, with lower expression levels showing increasing colder colors. Multiple upregulated DEGs (*Tbck*, *Reg3g*, *TRIM45*, *CXCR1*) and downregulated DEGs (*S100A11*, *reg3g*, *edil3*, *serpinb7*, *Gja5*, *Hspb1*, *Il17rc Tpd52l*) were each associated with either inflammatory- or barrier-regulatory pathways. (C) Bubble chart of KEGG pathways dysregulated in LPS/VILI-PBS-exposed rats with enrichment of inflammation-related pathways such as “cytokine–cytokine receptor interaction,” “viral interaction with cytokine,” and “IL-17 signaling” (see Table 1). The rich factor is the ratio of DEG numbers annotated for a specific pathway term to all gene numbers annotated in the pathway. The Q value is the corrected *P* value; the size of the bubble corresponds to the number of genes annotated in their respective pathway. The “cytokine–cytokine receptor interaction pathway” was the most significant LPS/VILI pathway (FDR 3.18E-09) consistent with previous reports in this LPS/VILI model.^29^ (D) Comparison of the number of genes annotated to specific KEGG pathway categories dysregulated between LPS/VILI-PBS and LPS/VILI- NTyP-100 rats. The x-axis represents the number of genes annotated to specific KEGG pathway categories, and the y-axis represents the KEGG pathway category. Despite the commonalities in dysregulated KEGG pathways, NTyP-100 treatment efficiently decreased the number of dysregulated genes contained in each pathway, with the majority of genes linked to human disease, metabolism, and innate immunity. Abbreviations: DEG, differentially expressed gene; FDR, false discovery rate; KEGG, Kyoto Encyclopedia of Genes and Genomes; LPS, lipopolysaccharide; MV, mechanical ventilation; PBS, phosphate-buffered saline.

**Table 1:** Pathway enrichment of dysregulated KEGG pathways in the LPS/VILI model.

KEGG terms	No. of observed genes	Total No. of genes contained	FDR
**Cytokine–cytokine receptor interaction**	27	259	3.18E-09
**Viral protein interaction with cytokine and cytokine receptor**	15	81	6.63E-08
**IL-17 signaling pathway**	15	86	9.21E-08
**Complement and coagulation cascades**	14	78	0.000000183
**Inflammatory mediator regulation of TRP channels**	11	100	0.0006
**Rheumatoid arthritis**	10	85	0.00075
**Amoebiasis**	10	92	0.0012
**Arachidonic acid metabolism**	9	78	0.0017
**Chagas disease**	10	100	0.0018
**Chemokine signaling pathway**	13	174	0.0022
**TNF signaling pathway**	10	107	0.0025
**Malaria**	7	49	0.0025
**Neuroactive ligand-receptor interaction**	17	334	0.0115
**Phenylalanine metabolism**	4	18	0.0172
**Linoleic acid metabolism**	5	35	0.0206
**JAK-STAT signaling pathway**	10	151	0.0223
**Calcium signaling pathway**	11	186	0.0286

Abbreviations: FDR, false discovery rate; KEGG, Kyoto Encyclopedia of Genes and Genomes; LPS, lipopolysaccharide; TRP, Transient receptor Potential; VILI, Ventilator Induced Lung Injury.

## Discussion

Current concepts of inflammatory vascular injury^1^ highlight the loss of EC barrier integrity and EC cytoskeletal remodeling, involving dynamic spatially defined nmMLCK-mediated MLC phosphorylation, increased actomyosin contractility, loss of cell-tethering junctional and extracellular matrix forces, and the increased formation of focal EC paracellular gaps.^18^^,^^20^^,^^21^ The ensuing vascular leakage is the pathophysiologic hallmark of severe critical care illnesses and multiple chronic inflammatory disorders. Unfortuna­tely, FDA-approved pharmacotherapies to address unremitting systemic and organ-specific vascular permeability, including the lung, remain a major unmet need.

Our prior efforts to gain mechanistic insights into the pathobiology of vascular leakage led to develop a barrier-regulatory model where nmMLCK-dependent increases in EC paracellular gap formation serve as a molecular mechanism underpinning vascular inflammatory responses^1^ including increased neutrophil diapedesis^34^ and vascular permeability.^18^^,^^20^^,^^21^ Supporting this model were studies utilizing genetically engineered mice with nmMLCK overexpression restricted to only the EC (*nmMylk*^-/-/ec-tg+^), which displayed significantly increased severity of LPS/VILI-induced lung injury.^20^^,^^21^ In contrast, global nmMLCK knockout mice (nmMLCK^-/-^) or the instillation of nmMLCK-silencing RNAs or the nmMLCK inhibitor, PIK, were strongly protective in preclinical LPS- and LPS/MV-induced inflammatory lung injury models with dramatic attenuation of lung permeability and leukocyte influx.^20^^,^^21^ The association of genetic variants in *MYLK* (the gene encoding nmMLCK), specifically 3 key coding single-nucleotide polymorphisms (SNPs; Pro21His, Ser147Pro, Val261Ala), further link nmMLCK preclinical studies to human ALI/ARDS pathobiology. These SNPs confer enhanced risk of inflammatory lung injury, ARDS severity, and mortality^35-37^ and are rare in European populations but quite common in individuals of African descent, an at-risk population for increased ARDS and sepsis severity.^2^^,^^36^^,^^38^ We have shown these *MYLK* coding SNPs to reduce nmMLCK protein translocation to the cell periphery, resulting in delayed EC gap closure and an extended duration of vascular leak.^39^

A critical quandary in the vascular permeability field is the paucity of insights into mechanisms of EC gap resealing that are needed to design permeability-mitigating therapeutic strategies to promote gap closure. Our laboratory was the first to report that a critical participant in the resolution phase of inflammation-induced vascular permeability is the Rac1 GTPase,^18^^,^^40^ which stabilizes the EC barrier and decreases stress fiber formation and barrier dysfunction. The DOCK1-ELMO1 complex is a critical regulator of Rac GTPases and lung EC barrier integrity and participates in the formation of lamellipodia containing nmMLCK, cortactin, focal adhesion components, and lamellipodin. Protruding lamellipodia facilitate paracellular gap closure and enhance vascular integrity^40^ (Figure 3B). Activation of the TLR4 signaling cascade markedly reduces DOCK1 and ELMO1 expression, influencing the severity of inflammatory lung injury in ARDS/VILI models.^40^

Our laboratory was the first to report that the naturally occurring bioactive sphingolipid S1P functions as a potent angiogenic factor^5^ and profoundly enhances vascular barrier integrity both in vitro and in vivo.^6^^,^^41^ Subsequent studies detailed the ligation of the G protein–coupled S1PR1 by S1P or barrier-protective S1P analogues to rapidly initiate a Rac GTPase-dependent signaling cascade that drives peripheral EC cytoskeletal remodeling and lamellipodia formation in a biophysically and spatially defined manner with the essential requirement for posttranslational modifications of key cytoskeletal genes^35^^,^^42-44^ to increase EC barrier integrity and decrease vascular permeability in vitro.^4^^,^^6^^,^^11^^,^^18^^,^^24^^,^^45^^,^^46^ In vivo studies corroborated the utility of S1P and S1P analogues as barrier-protective strategies.^6^^,^^47-49^ Our recent biophysical studies using time-lapse optical imaging and atomic force microscopy demonstrated S1P to significantly enhance EC motility and increase the amplitudes of lamellipodia protrusion into the paracellular space to increase rates of EC gap closure.^50^ Importantly, as noted earlier, the utility of S1P and specific S1P analogues (SEW-2871, FTY-720), as a viable therapeutic strategies to reduce vascular leak,^8^^,^^9^ is greatly reduced by their shared capacity to reduce S1PR1 expression via the induction of β-arrestin recruitment, S1PR1 ubiquitination, and S1PR1 proteasomal degradation^11^^,^^14^—events that potentially result in significant EC barrier compromise and exaggerated permeability responses to edemagenic agents.^4^^,^^6^^,^^13^^,^^14^

The current study employed a novel nanocarrier (NTyP-100) conjugated to the phosphonated S1P/FTY-720 analogue, tysiponate (TySIP), that neither induces S1PR1 protein degradation^16^^,^^24^ or promotes the immunosuppressive properties observed with FTY-720.^16^^,^^24^ TySIP is, however, highly effective in enhancing lung vascular integrity and reducing vascular permeability in preclinical models of ventilator-, bleomycin-, LPS-, and methicillin-resistant *Staphylococcus aureus* (MRSA)–induced lung injury.^17^^,^^41^^,^^47^^,^^48^ Consistent with the nmMLCK-regulated EC cytoskeleton as a high impact druggable target, we encargoed the novel NTyP-100 nanocarrier with the nmMLCK peptide inhibitor, PIK, as a complementary strategy to potentially synergize with TySIP to halt vascular leakage and restore vascular barrier integrity. This strategy produced highly significant reductions in ALI severity (∼40% reduction) reflected by decreased H&E staining, reduced levels of BAL cells/protein, and attenuated EBD accumulation accompanied by decreased p-MLC immunoreactivity, reflecting PIK effectiveness in inhibiting nmMLCK activation to promote vascular barrier protection.

A key finding of our study was the observation that the functional effects of the NTyP-100 nanocarrier to clearly extend beyond the immediate effects on EC cytoskeletal remodeling and vascular barrier restoration. Biochemical and genomic indices in the 2-hit LPS/VILI model were highly consistent with similar LPS/VILI genomic studies in rats^27^^,^^29^ with dysregulation of innate immunity pathways (cytokine–cytokine receptor interaction, viral interaction with cytokine, IL-17 signaling, ROS). Importantly, LPS/VILI-exposed rats receiving the NTyP-100 therapy showed abolishment of LPS/VILI-mediated upregulation of NF-κB-, MAPK signaling-, and ROS-related genes, findings consistent with previously described influence of both S1P and nmMLCK on key biologic pathways.^23^^,^^49^ Multiple NTyP-100–mediated upregulated DEGs included *Reg3γ*, known to influence systemic and vascular inflammation^51^; *EDIL3*, which binds to specific integrin receptors to influence vascular integrity^52^; and *TRIM45*, which is an E3 ubiquitin ligase regulating the NLRP3 inflammasome, NF-κB signaling, and pyroptosis. NTyP-100–driven downregulated DEGs included *SerpinB7*, a serine protease inhibitor that participates in skin barrier function and inflammatory responses,^53^ and *ACKR1* (atypical chemokine receptor 1, also known as the Duffy antigen receptor for chemokines), which plays a multifaceted role in regulating inflammation and leukocyte recruitment and extravasation within the vasculature.^54^ In addition, we noted upregulation of fibroblast activation/proliferation genes in LPS/VILI-exposed rats that reverted their dysregulated status after NTyP-100 administration. The observed upregulation of genes centrally involved in development of organ fibrosis (*Tgfb1*, *Pdgfrb/c*, *Cdk4*, *Esr1*) is of interest in light of recent human ARDS molecular phenotyping studies showing collagen production and fibrosis genes to predict ARDS mortality.^55^

Current strategies to enhance vascular barrier function include endogenous EC barrier enhancers (in addition to S1P),^6^ such as angiopoietin 1 (ANGPT1)^56^ and hepatocyte growth factor.^12^ Additional strategies to mitigate vascular barrier dysfunction include inhibitors of vascular endothelial growth factor,^57^ originally known as vascular permeability factor,^58^ and the barrier-destabilizing angiopoietin 2 (ANGPT2), although an ANGPT2-blocking mAb failed to reduce the number of ventilator days in hospitalized patients with COVID-19 pneumonia.^59^ Statins are primarily known for their cholesterol-lowering effects but also reduce inflammation in the vascular system and modulate vascular permeability,^18^^,^^60^^,^^61^ including upregulation of S1PR1.^62^ Imatinib, while primarily known for its role as a cancer treatment, reduces vascular leak in preclinical ALI but with complex positive and negative effects.^26^^,^^63^ Strategies to neutralize the P-selectin ligand, PSGL1, critically involved in PMN trafficking and EC barrier dysfunction and encoded by *SELPLG*, an ARDS susceptibility gene,^2^ include the FDA-approved P-selectin-binding mAb, crizanlizumab, and TSGL-Ig, a recombinant tandem PSGL1 immunoglobulin fusion molecule.^64^ Our encouraging results with NTyP-100 administration to rats with LPS/VILI-induced vascular leak should be viewed in the context of the multitude of failed phase 2/3 ARDS therapeutic trials that can be attributed to overreliance on murine LPS models without MV exposure.

In summary, the current study reports the evaluation of the novel NTyP-100 nanocarrier conjugated to the nonhydrolysable S1P analogue and the selective S1PR1 receptor agonist TySIP, with the nmMLCK inhibitory peptide, PIK, as cargo. Despite study limitations that include performing all molecular assessments at a single time point, the limitation of bulk RNA-seq, and the use of a single concentrations of each reagent tested, the NTyP-100 nanocarrier proved to be a highly effective barrier-enhancing strategy and represents the platform of combining 2 complementary EC-barrier enhancing strategies into a single nanocarrier allows targeting of S1PR1 in the vasculature. The NTyP-100 nanocarrier platform utilized may potentially allow for lower dosing of encargoed EC barrier-enhancing or inflammation-reducing therapeutic candidates with complementary mechanisms of action. As vascular barrier disruption is a cardinal feature of multiple acute and chronic inflammatory states/disorders with accompanying organ edema/injury, NTyP-100 may prove a viable approach to address the unmet need for novel therapeutics that mitigate inflammatory injury and reduce vascular permeability.

## Supplementary Material

aanag069_Supplementary_Data

## Data Availability

The RNA-seq datasets generated and analyzed during the current study will be deposited in the Sequence Read Archive at the National Center for Biotechnology Information.
